# Determination of the most effective design for the measurement of photosynthetic light-response curves for planted *Larix olgensis* trees

**DOI:** 10.1038/s41598-020-68429-w

**Published:** 2020-07-15

**Authors:** Qiang Liu, Weiwei Jia, Fengri Li

**Affiliations:** 1 Key Laboratory of Sustainable Forest Ecosystem Management-Ministry of Education, School of Forestry, Northeast Forestry University, Harbin, 150040 Heilingjiang People’s Republic of China; 2 School of Forestry, Hebei Agricultural University, Baoding, 071001 Hebei People’s Republic of China

**Keywords:** Forestry, Light responses

## Abstract

A photosynthetic light-response (PLR) curve is a mathematical description of a single biochemical process and has been widely applied in many eco-physiological models. To date, many PLR measurement designs have been suggested, although their differences have rarely been explored, and the most effective design has not been determined. In this study, we measured three types of PLR curves (High, Middle and Low) from planted *Larix olgensis* trees by setting 31 photosynthetically active radiation (PAR) gradients. More than 530 million designs with different combinations of PAR gradients from 5 to 30 measured points were conducted to fit each of the three types of PLR curves. The influence of different PLR measurement designs on the goodness of fit of the PLR curves and the accuracy of the estimated photosynthetic indicators were analysed, and the optimal design was determined. The results showed that the measurement designs with fewer PAR gradients generally resulted in worse predicted accuracy for the photosynthetic indicators. However, the accuracy increased and remained stable when more than ten measurement points were used for the PAR gradients. The mean percent error (M%E) of the estimated maximum net photosynthetic rate (*P*_max_) and dark respiratory rate (*R*_d_) for the designs with less than ten measurement points were, on average, 16.4 times and 20.1 times greater than those for the designs with more than ten measurement points. For a single tree, a unique PLR curve design generally reduced the accuracy of the predicted photosynthetic indicators. Thus, three optimal measurement designs were provided for the three PLR curve types, in which the root mean square error (RMSE) values reduced by an average of 8.3% and the coefficient of determination (R^2^) values increased by 0.3%. The optimal design for the High PLR curve type should shift more towards high-intensity PAR values, which is in contrast to the optimal design for the Low PLR curve type, which should shift more towards low-intensity PAR values.

## Introduction

The photosynthetic light-response (PLR) curve reflects the instantaneous response of the net photosynthetic rate (*P*_n_) to different gradients of photosynthetically active radiation (PAR). It can provide measures of many photosynthetic indicators, such as the maximum *P*_n_ (*P*_max_), dark respiration rate (*R*_d_), apparent quantum yield (AQY), light compensation point (LCP) and light saturated point (LSP), for analysing plant photosynthetic activity^1^. In addition, it is also a basic element for modelling the photosynthesis^2–4^ and primary productivity^5–7^ of vegetation and forests. In recent studies, the application of the PLR model has been expanded from a single leaf to larger scales by linking some leaf functional traits and environmental conditions, such as the leaf mass per area (LMA), nitrogen (N) content^8–10^, leaf temperature (*T*_leaf_) and global site factor (GSF)^11–13^. Our previous study successfully established a dynamic crown PLR model for *Larix olgensis* trees by linking the LMA, *T*_leaf_, vapour pressure deficit (VPD) and relative depth into the crown (RDINC) in the original PLR equation^13^. These results laid the foundation for estimating the net primary production (NPP) and further exploring its allocation mechanisms in individual *L. olgensis* trees.

Although the PLR model has been widely applied, the questions that most frequently confuse researchers during the design of measurements are as follows: how many PAR gradients and what specific PAR values should be chosen to ensure the best design? Table 1 lists the many designs that have been used to measure the PLR curves in different studies. Although some studies used the same number of PAR gradients, the specific PAR values differed. However, none of these studies explained why such designs were selected. Thus, how the different designs will affect the goodness of fit of the PLR curves and the results of the predicted accuracy of the estimated photosynthetic indicators remain unclear.

**Table 1:** Summary of the different PLR designs in part of other researches.

Species	Number	Specific PAR gradients of PLR curves
*Oryza sativa*^10,19^	16	0, 50, 100, 150, 200, 400, 600, 800, 1,000, 1,200, 1,400, 1,600, 1,800, 1900, 1950, 2000
	15	0, 25, 50, 100, 150, 200, 300, 400, 600, 800, 1,000, 1,200, 1,400, 1,600, 2000
*Boswellia papyrifera*^20^	14	0, 50, 100, 200, 300, 400, 600, 800, 1,000, 1,200, 1,400, 1,600, 1,800, 2000
*Nicotiana tabacum*^21^	13	0, 20, 50, 80, 100, 200, 400, 600, 800, 1,000, 1,200, 1,500, 1,800
*Populus balsamifera* × *Populus trichocarpa*; *Populus maximowiczii* × *Populus balsamifera*^22^	13	0, 50, 100, 300, 400, 500, 600, 700, 800, 900, 1,000, 1,600, 2000
*Boswellia papyrifera*;* Capsicum annuum*; *Koelreuteria paniculata*;* Zea mays*;* Sorghum bicolor*^23^	13	0, 50, 100, 200, 400, 600, 800, 1,000, 1,200, 1,400, 1,600, 1,800, 2000
*Acer saccharum*^4^	12	0, 100, 200, 400, 600, 800, 1,000, 1,200, 1,400, 1,600, 1,800,2000
*Larix olgensis*;* Larix kaempferi*^24^	13	0, 30, 80, 120, 160, 200, 400, 600, 800, 1,000, 1,200, 1,400, 1,600
*Populus trichocarpa* × *Populus deltoids*;* Populus trichocarpa*;* Populus nigra*^25^	12	0, 25, 50, 75, 100, 200, 300, 400, 500, 1,000, 1,500, 2000
*Pinus cembra*^26^	11	0, 50, 100, 200, 300, 400, 500, 750, 1,000, 1,500, 2000
25 Herbaceous species^8^	10	0, 50, 100, 200, 300, 400, 600, 800, 1,000, 1,310
*Zea mays*^27^	10	0, 50, 100, 200, 300, 500, 700, 1,000, 1,500, 2000
*Larix gmelinii*^28^	10	0, 50, 100, 150, 400, 800, 1,200, 1,500, 2000
*Ficus insipida*;* Castilla elastica*^29^	9	0, 50, 100, 250, 500, 750, 1,000, 1,500, 2000
*Juglans regia*^30^	9	0, 25, 50, 100, 250, 500, 1,000, 1,500, 2000
*Nothofagus cunninghamii*^31^	9	0, 20, 50, 100, 200, 500, 1,000, 1,500, 2000
*Kalmia angustifolia*^32^	9	0, 10, 50, 100, 200, 300, 400, 600, 800
*Pinus ponderosa*;* Ceanothus cordulatus*;* Arctostaphylos manzanita*^3^	8	10, 50, 100, 200, 500, 800, 1,200, 1,800
*Pseudotsuga menziesii*;* Tsuga heterophylla*^33^	8	10, 50, 100, 200, 400, 800, 1,400, 2000
*Populus tremuloides*;* Abies lasiocarpa*^34^	8	0, 50, 100, 200, 500, 1,000, 1,500, 2000
*Castanea dentate*^35^	8	0, 50, 100, 200, 500, 800, 1,200, 1,600
*Quercus douglasii*^36^	7	50, 100, 200, 400, 600, 1,000, 1,400
*Fagus crenata*^37^	7	0, 50, 100, 200, 400, 700, 1,000
*Helianthus annus*^38^	7	10, 50, 100, 200, 500, 1,500, 2000
*Quercus pngodci*^39^	6	0, 50, 150, 300, 800, 1,800
25 Herbaceous species^9^	5	75, 150, 175, 500, 700

The PLR curves for trees generally exhibit spatial variations, even within an individual crown^14–16^. Thus, it is inappropriate to measure only one position to represent the photosynthetic characteristics of the whole crown^13^. Our previous study^13,17,18^ suggested that the PLR curves significantly differed in the vertical direction in the crown of a *L. olgensis* tree. More samples must be selected to analyse the PLR curves throughout tree crowns, especially for tall trees. However, more samples will consequently require more time on a single tree, which will limit the amount of data collected over a large scale. Thus, an efficient and scientific design for measuring PLR curves is necessary and urgent for analysing photosynthetic characteristics, especially for trees.

*Larix olgensis* accounts for 36% of the total area of all plantations in northeastern China, which indicated that it is the main tree species used for afforestation. The objectives of this investigation are (1) to analyse the influence of different designs on the goodness of fit of PLR curves; (2) to further analyse the influence of different designs on the predicted accuracy for the estimated photosynthetic indicators and (3) to propose an optimal design for the measurement of PLR curves for planted *L. olgensis* trees.

## Materials and methods

### Site description

The experiments were conducted in 2017 at the experimental forest farm of Northeast Forestry University in Maoershan (45° 23′ 21″ N, 127° 37′ 56″ E). The site is characterized by a midlatitude monsoon climate, with warm, wet summers and cold, dry winters. The average temperature throughout the growing season at the site is 17.0 °C (with a range from − 1.3 to 39.4 °C), the average precipitation throughout the growing season is 944 mm. The type of soil is typic Eutroboralfs, and the total forest coverage is approximately 83.3%, including 14.7% plantation.

### PLR measurements

**Figure 1:**
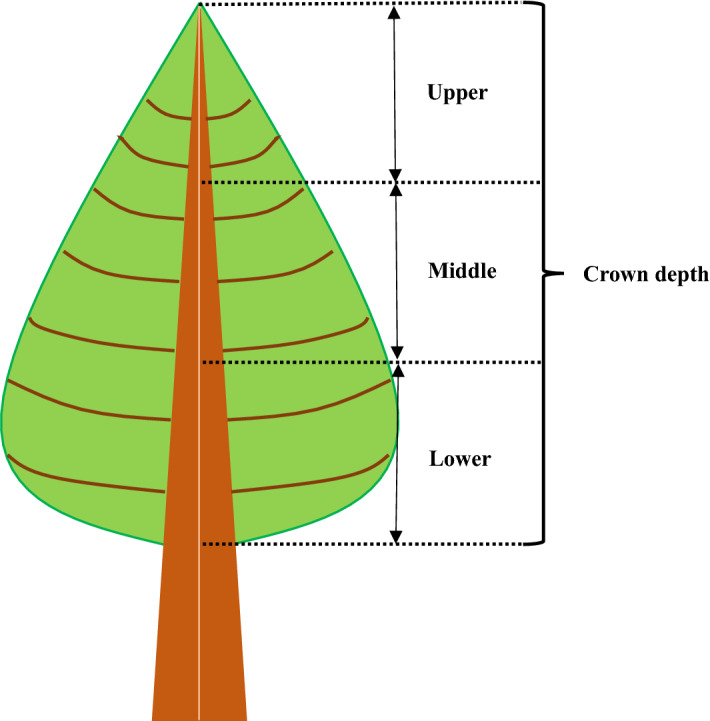
Sketch map of the crown divisions. Upper, Middle and Lower represent three equal divisions of crown depth in the vertical direction.

**Figure 2:**
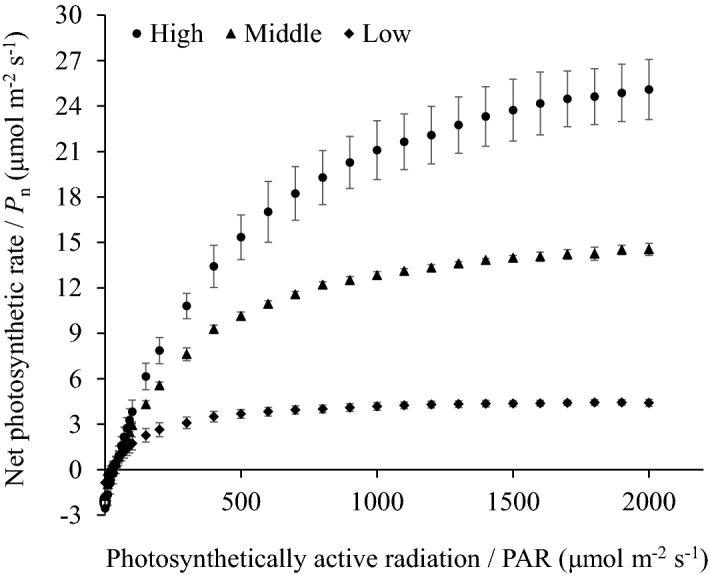
Summary of the photosynthetic light response (PLR) curves of *Larix olgensis* trees. High, Middle and Low represent three typical PLR curves that were measured in the Upper, Middle and Lower positions within the crown, respectively.

In this study, three sample plots (20 m × 30 m) were established within 18-year-old pure *L. olgensis* plantations of the same habitat. The diameter at breast height (DBH) and tree height (H) were measured for each tree whose DBH was greater than 5 cm in each plot, and the quadratic mean diameters (Dg) for three plots were calculated independently. Then, three sample trees with DBH values respectively similar to the Dg of the three plots were selected to represent the average state of each plot. According to previous research^13,17,18^, the upper limit of the PLR curves was significantly different within different crown whorls in the vertical direction. Thus, we divided the crowns of three sample trees respectively into three equal divisions based on the crown depth (Fig. 1). Three types of PLR curves, which were tagged as High, Middle and Low (Fig. 2), were measured in each division (Upper, Middle and Lower) for a sample tree. The measurements were conducted using a portable photosynthesis system (LI-6400XT, LI-COR, Inc., Lincoln, Nebraska, USA) coupled with a standard light-emitting diode (LED) light source (Li-6400-02B) at 31 PAR levels (2,000, 1,900, 1,800, 1,700, 1,600, 1,500, 1,400, 1,300, 1,200, 1,100, 1,000, 900, 800, 700, 600, 500, 400, 300, 200, 150, 100, 90, 80, 70, 60, 50, 40, 30, 20, 10 and 0 μmol m^−2^ s^−1^). As needle clusters generally overlapped each other, those covered needles could not receive light; therefore, they only have respiration but no photosynthesis. If we do not remove these needles, then they will be calculated into the sample leaf area and consequently reduce the value of the PLR curves. Therefore, the covered needles were removed before measuring to avoid incorrect PLR curve measurements. The reserved sample needles were acclimated for 20 min at a CO_2_ concentration of 370 μmol m^−2^ s^−1^ and a PAR value of 1,400 μmol m^−2^ s^−1^. Then, the sample needles were allowed to equilibrate for a minimum of 2 min at each PAR gradient before the data were logged during the measurement of the PLR curves. The PLR curves were measured from 8:00 to 17:00 from the 25th of August to the 27th of August in 2018. The temperature (T) and relative humidity (RH) were approximately 28–30 ℃ and 30–40% during the measurement, which would not cause stomatal closure. Once the measurements of the PLR curves were performed, the sample needles were scanned and surveyed with Image-Pro Plus 6.0 software (Media Cybernetics, Bethesda, MD, USA) in the laboratory, resulting in a projected leaf area. These methods expand upon those given in previous publications^13,17^.

### Model descriptions

In this study, the PLR curves were fitted with the modified Mitscherlich equation^13^:

1 $$ P_{{\text{n}}} = P_{\max } \times \left( {1 - {\text{e}}^{{\left( { - {\text{AQY}} \times {\text{PAR}}/P_{\max } } \right)}} } \right) - R_{{\text{d}}} $$

where *P*_max_ is the maximum net photosynthetic rate (μmol m^−2^ s^−1^), AQY is the apparent quantum yield, and *R*_d_ is the dark respiration rate (μmol m^−2^ s^−1^).

### Model assessment and validation

The assessment of the PLR curve model was based on the root mean square error (RMSE, Eq. 2) and the coefficient of determination (R^2^, Eq. 3). The predicted accuracies of the photosynthetic indicators were evaluated by the mean error (ME, Eq. 4) and the mean percent error (M%E, Eq. 5) as follows:

2 $$\mathrm{R}\mathrm{M}\mathrm{S}\mathrm{E}=\sqrt{\frac{\sum_{{\varvec{i}}=1}^{{\varvec{n}}}{\left({{\varvec{y}}}_{{\varvec{i}}}-{\widehat{{\varvec{y}}}}_{{\varvec{i}}}\right)}^{2}}{{\varvec{n}}-{\varvec{p}}}}$$

3 $${\mathrm{R}}^{2}=1-\frac{\sum_{{\varvec{i}}=1}^{{\varvec{n}}}{\left({{\varvec{y}}}_{{\varvec{i}}}-{\widehat{{\varvec{y}}}}_{{\varvec{i}}}\right)}^{2}}{\sum_{{\varvec{i}}=1}^{{\varvec{n}}}{\left({{\varvec{y}}}_{{\varvec{i}}}-\stackrel{-}{{\varvec{y}}}\right)}^{2}}$$

4 $$\mathrm{M}\mathrm{E}=\sum_{i=1}^{n}\left(\frac{{y}_{i}-{\widehat{{\varvec{y}}}}_{{\varvec{i}}}}{n}\right)$$

5 $$\mathrm{M}\mathrm{\%}\mathrm{E}=\frac{1}{n}\sum_{i=1}^{n}\left(\frac{{y}_{i}-{\widehat{{\varvec{y}}}}_{{\varvec{i}}}}{yi}\right)\times 100\boldsymbol{\%}$$

where y_i_ is the observed value; $\stackrel{-}{{\varvec{y}}}$ is the mean of the observed values; ${\widehat{{\varvec{y}}}}_{{\varvec{i}}}$ is the predicted value; n is the number of observations; and *p* is the number of parameters.

### Determination of the optimal design for measuring the PLR curve

The 31 observed *P*_n_ values corresponding to 31 PAR gradients in each PLR curve were classified into 26 groups based on the number of PAR gradients from 5 to 30, in which 0 and 2000 were fixed points. In each group, the method of non-repetitive random sampling were used to ensure that all the combinations were considered and all the sampling designs were listed in Table 2. In total, there were more than 530 million combinations of PAR gradients to be fitted by using the “dplyr” package in R software^40^, and the whole fitting process took more than 200 h. Then, the best combination with the smallest RMSE value and largest R^2^ value in each group was selected. Thereafter, the predicted accuracies of the estimated parameters, such as *P*_max_, AQY and *R*_d_, which represent the important photosynthetic indicators, were evaluated by the ME and M%E. Finally, the most effective design for PLR measurement was determined by considering the minimum measured PAR gradients based on the premise of ensuring acceptable accuracy of the estimated photosynthetic indicators.

**Table 2:** Summary of the sampling designs.

Number of method points of PAR	Number of combinations	Number of method points of PAR	Number of combinations
5	${\mathrm{C}}_{29}^{3}=3654$	18	${\mathrm{C}}_{29}^{16}=67863915$
6	${\mathrm{C}}_{29}^{4}=23751$	19	${\mathrm{C}}_{29}^{17}=51895935$
7	${\mathrm{C}}_{29}^{5}=118755$	20	${\mathrm{C}}_{29}^{18}=34597290$
8	${\mathrm{C}}_{29}^{6}=475020$	21	${\mathrm{C}}_{29}^{19}=20030010$
9	${\mathrm{C}}_{29}^{7}=1560780$	22	${\mathrm{C}}_{29}^{20}=10015005$
10	${\mathrm{C}}_{29}^{8}=4292145$	23	${\mathrm{C}}_{29}^{21}=4292145$
11	${\mathrm{C}}_{29}^{9}=10015005$	24	${\mathrm{C}}_{29}^{22}=1560780$
12	${\mathrm{C}}_{29}^{10}=20030010$	25	${\mathrm{C}}_{29}^{23}=475020$
13	${\mathrm{C}}_{29}^{11}=34597290$	26	${\mathrm{C}}_{29}^{24}=118755$
14	${\mathrm{C}}_{29}^{12}=51895935$	27	${\mathrm{C}}_{29}^{25}=23751$
15	${\mathrm{C}}_{29}^{13}=67863915$	28	${\mathrm{C}}_{29}^{26}=3654$
16	${\mathrm{C}}_{29}^{14}=77558760$	29	${\mathrm{C}}_{29}^{27}=406$
17	${\mathrm{C}}_{29}^{15}=77558760$	30	${\mathrm{C}}_{29}^{28}=29$
–	–	Total	536,870,475

## Results

### Performance of different PLR measurement designs

**Figure 3:**
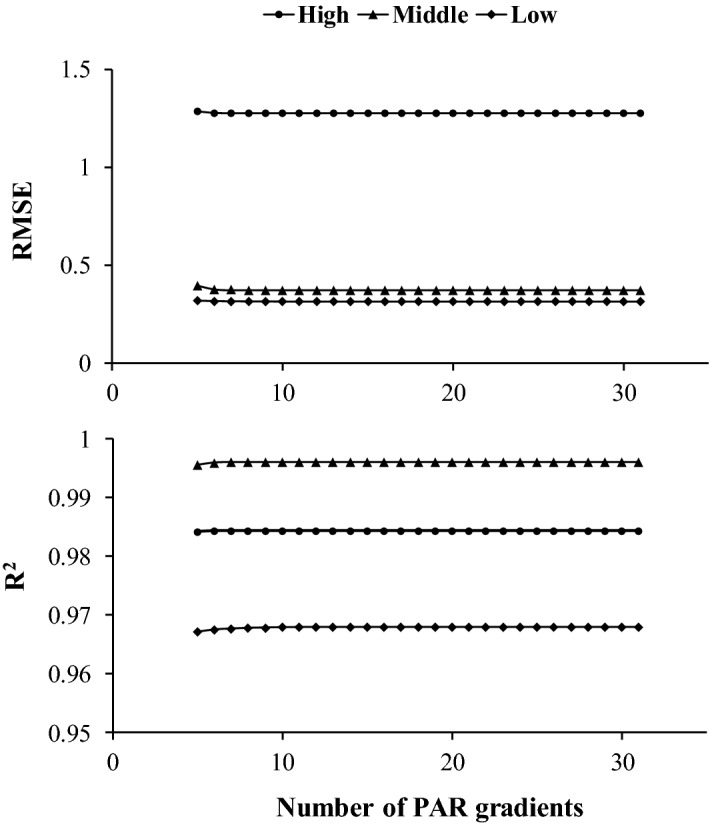
Root mean square error (RMSE) and the coefficient of determination (R^2^) of different designs for measuring the three types of photosynthetic light response (PLR) curves. High, Middle and Low represent three typical PLR curves that were measured in the Upper, Middle and Lower positions within the crown, respectively. PAR represents photosynthetically active radiation.

The goodness of fit (RMSE and R^2^) of all the designs with different combinations of PAR gradients in each group were calculated, and the corresponding values for the best combination are shown in Fig. 3. The results showed that the RMSE values in the Low and Middle PLR curve types were, on average, 75.4% and 70.7% smaller than those in the High PLR curve type; the R^2^ values in the Middle PLR curve type were greater than those in the High and Low PLR curve types, although they were, on average, 0.012 and 0.028 greater. The performance of the fitting results became stable when the number of PAR gradients was more than 5.

### Influence of the different PLR measurement designs on the estimated photosynthetic indicators

**Figure 4:**
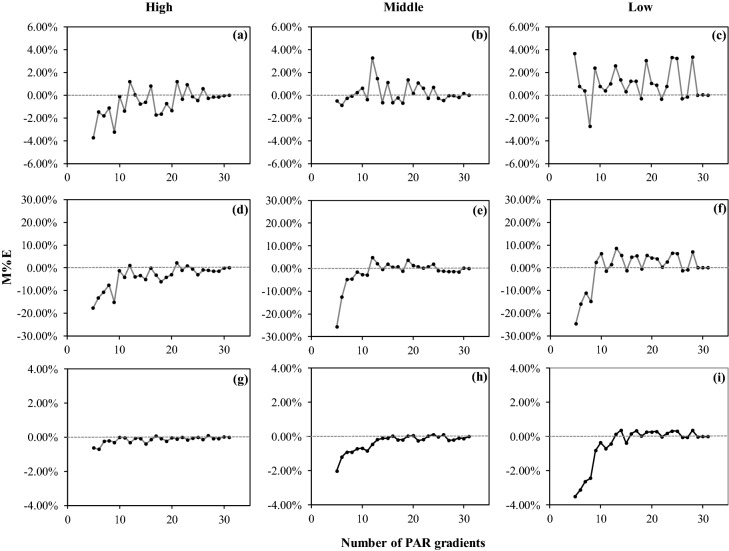
Mean percent error (M%E) of the estimated photosynthetic indicators by different designs for measuring the three types of photosynthetic light response (PLR) curves (High, Middle and Low represent three typical PLR curves that were measured in the Upper, Middle and Lower positions within the crown, respectively): (**a**–**c**) M%E of the apparent quantum yield (AQY); (**d**–**f**) M%E of the dark respiration rate (*R*_d_); and (**g**–**i**) M%E of the maximum net photosynthetic rate (*P*_max_). PAR represents photosynthetically active radiation.

Figure 4 shows the influence of the different PLR measurement designs on the estimated photosynthetic indicators (AQY, *R*_d_ and *P*_max_). The M%E values for estimating AQY in all three types of PLR curves were small and fluctuated between 4 and − 4% (Fig. 4a–c). The estimated AQY of the High PLR curve type was overestimated on average (Fig. 4a), but the estimated AQY of the Low PLR curve type was underestimated on average (Fig. 4c). The absolute M%E values of the estimated AQY for the designs with less than ten measured points were, on average, 9.5 times greater than the values for those with more than ten measured points. The M%E values for estimating *R*_d_ were lowest when the number of PAR gradients was set to five regardless of the High, Middle or Low PLR curve types (− 18%, − 26% and − 25%, Fig. 4d–f); then, the M%E values remained between 10 and − 10% in all types of PLR curves when the number of measured points for the PAR gradients was more than 10. The estimated *R*_d_ values were overestimated in all three types of PLR curves (Fig. 4d–f) when the number of measured points for the PAR gradients was less than 10. However, the estimated *R*_d_ exhibited a similar regulation as the AQY, which was overestimated for the High PLR curve type (Fig. 4d) and underestimated for the Low PLR curve type (Fig. 4f) when the number of measured points of the PAR gradients was more than 10. The absolute M%E values of the estimated *R*_d_ for the designs with less than ten measured points were, on average, 6.7 times greater than the values for the designs with more than ten measured points. The M%E values for estimating *P*_max_ were lower when less than ten measured points were used for the PAR gradients, indicating that the *P*_max_ values were overestimated on average. Then, these values remained between 1 and − 1% for all types of PLR curves until more than ten measured points for the PAR gradients were used. The absolute M%E values of the estimated *P*_max_ values for the designs with fewer than ten measured points were, on average, 4.8 times greater than the values for the designs with more than ten measured points.

### Determination of the optimal measuring design of the PLR curve

**Figure 5:**
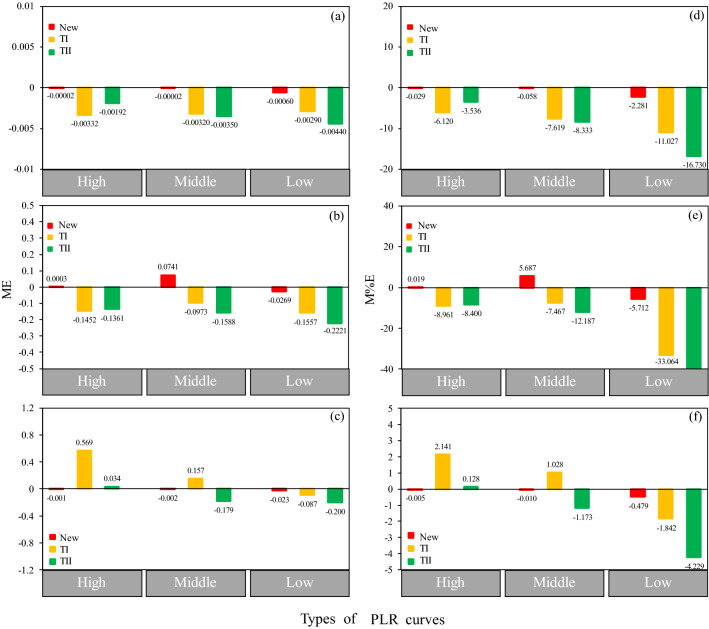
Comparison of the performance of the estimated photosynthetic indicators between our new design and the two other designs for the three types of photosynthetic light response (PLR) curves (High, Middle and Low represent three typical PLR curves that were measured in the Upper, Middle and Lower positions within the crown, respectively): (**a**–**c**) mean error (ME) values of the apparent quantum yield (AQY), dark respiration rate (*R*_d_) and maximum net photosynthetic rate (*P*_max_); and (**d**–**f**) mean percent error (M%E) values of AQY, *R*_d_ and *P*_max_.

10 PAR gradients achieved good performance for the PLR fitting and parameter estimations (Fig. 4) according to the above results. Thus, we determined the optimal measurement design by contrasting the performance of all combinations of the 10 PAR gradients in the three types of PLR curves (Table 3). In addition, we also evaluate the performance between our new measurement design and the other designs for the same Larch species^24,28^ based on the goodness of fit (Table 3). The results showed that the optimal designs for measuring the three types of PLR curves were different. For the High PLR curve type, the PAR gradients shifted more towards high PAR values, but for the Low PLR curve type, the PAR gradients shifted more towards low PAR values. Our new designs performed better than the other designs in all three types of PLR curves, with increased R^2^ values and decreased RMSE values. The performances of the estimated photosynthetic indicators were compared between our new design and the two other designs for the three types of PLR curves (Fig. 5). Significant difference of the accuracy for parameter estimations between our new design and the two other designs was appeared (Table 4). The results showed that the AQY and *P*_max_ values were generally overestimated in all three types of PLR curves by our new design (Fig. 5a,c,d,f). The *R*_d_ values were also overestimated in the Low PLR curve type but underestimated in the High and Middle PLR curve types (Fig. 5b,e). The AQY and *R*_d_ values from the TI design were obviously overestimated (Fig. 5a,b,d,e), and the *P*_max_ values were obviously underestimated in the High and Middle PLR curve types but overestimated in the Low PLR curve type (Fig. 5c,f). In addition, the estimated AQY, *R*_d_ and *P*_max_ values also exhibited greater differences when using the TII design than those exhibited when using our new design. In summary, our new design provided better estimations for AQY, *R*_d_ and *P*_max_ values in all three types of PLR curves.

**Table 3:** Comparison between the new optimal measurement designs and other measurement designs of three types of PLR curves.

Type	Designs	Sampling of PAR (μmol m^−2^ s^−1^)	Parameters		
			RMSE	R^2^	Chi-squared value
High	New	0, 40, 80, 90, 150, 900, 1,000, 1,200, 1,700, 2000	1.2785	09,844	0.1199
	TI^24^	0, 30, 80, 120, 160, 200, 400, 600, 800, 1,000, 1,200, 1,400, 1,600	1.3069	0.9837	0.1405
	TII^28^	0, 50, 100, 150, 400, 800, 1,200, 1,500, 2000	1.2823	0.9843	0.1274
Middle	New	0, 80, 90, 100, 300, 600, 700, 1,000, 1,600, 2000	0.3744	0.9960	0.0390
	TI^24^	0, 30, 80, 120, 160, 200, 400, 600, 800, 1,000, 1,200, 1,400, 1,600	0.4087	0.9953	0.0748
	TII^28^	0, 50, 100, 150, 400, 800, 1,200, 1,500, 2000	0.3976	0.9955	0.0676
Low	New	0, 30, 50, 60, 70, 80, 300, 400, 1,500, 2000	0.3149	0.9680	0.1815
	TI^24^	0, 30, 80, 120, 160, 200, 400, 600, 800, 1,000, 1,200, 1,400, 1,600	0.3206	0.9668	0.1923
	TII^28^	0, 50, 100, 150, 400, 800, 1,200, 1,500, 2000	0.3222	0.9665	0.1807

New is our optimal measurement design for the PLR curve; and TI and TII are another two designs in different papers for the same larch species (Table 1).

**Table 4:** ANOVA results for the accuracy of parameter estimations between the new optimal measurement designs and other measurement designs of three types of PLR curves.

	ME			M%E		
	*P*_max_	AQY	*R*_d_	*P*_max_	AQY	*R*_d_
New	− 0.008 + 0.012^b^	− 0.0002 + 0.0003^b^	0.016 + 0.052^b^	− 0.165 + 0.272^b^	− 0.790 + 1.292^b^	− 0.002 + 5.70^b^
TI	− 0.115 + 0.129^a^	− 0.0031 + 0.0002^a^	− 0.133 + 0.031^a^	0.442 + 2.055^c^	− 8.255 + 2.515^a^	− 16.498 + 14.367^a^
TII	0.213 + 0.331^c^	− 0.0033 + 0.0013^a^	− 0.172 + 0.045^a^	− 1.758 + 2.237^a^	− 9.533 + 6.678^a^	− 22.584 + 21.372^a^

New is our optimal measurement design for the PLR curve; and TI and TII are another two designs in different papers for the same larch species (Table 1). Different superscripts mean significant difference.

## Discussion

The PLR curve is an important semiempirical model for describing the response of the *P*_n_ to PAR, and it has been frequently applied as an effective tool for identifying a series of photosynthetic indicators^1^ and a basic element for modelling photosynthesis^2–4^ and calculating the NPP^5–7^. To date, there have been many different designs for measuring PLR curves in different species or even in the same species (Table 1). However, it remains unclear why such designs are used and which design is the most accurate and effective. During the measurement of the PLR curve, each additional PAR gradient will increase the measurement time by at least three minutes, which indicates that the measurement time for a PLR curve can be reduced by at least 33 min if the number of PAR gradient measurements are reduced from 16 to 5 for only one sample leaf. This result is of great significance for the formulation of photosynthetic measurement schemes for trees because the PLR curves for trees generally exhibit significant vertical variations throughout a crown^13–16^ (Fig. 2), which indicates that more samples must be selected from different crown positions when modelling the PLR curve of a crown. Thus, an effective design with fewer PAR gradients that can shorten the measurement time for a single sample leaf will directly affect the size of a dataset.

The special PAR gradients that are frequently chosen in most designs are 0 μmol m^−2^ s^−1^ and 2000 μmol m^−2^ s^−1^ (Table 1) because the *R*_d_ is a specific *P*_n_ value when PAR is 0 μmol m^−2^ s^−1^ and the peak of PAR on a sunny day is approximately 2000 μmol m^−2^ s^−1^. In previous studies, the number of PAR gradients for PLR curve measurement was at least 5, although most of these gradients included more than seven points and the greatest number of PAR gradients was 17^13,18^ (Table 1). However, few studies have explored the difference between different PLR measurement designs. In this study, we analysed the performance of all PLR curve measurement designs with the number of PAR gradients ranging from 5 to 31. The results showed that the fitting results remained relatively stable when more than 5 PAR gradients were used (Fig. 3). The accuracies of the estimated photosynthetic indicators (AQY, *R*_d_ and *P*_max_) were worse when there were fewer PAR gradients. However, the accuracy increased when the number of PAR gradients was set to more than 10 (Fig. 4), indicating that 10 PAR gradients may be the most effective design as the measurement of 10 PAR gradients requires relatively little time while ensuring acceptable accuracy for the estimation photosynthetic indicators. The accuracies of the estimated AQY, *R*_d_ and *P*_max_ values were more stable for the Middle PLR curve type than those for the High and Low PLR curve types, and the accuracies were more stable for the Middle PLR curve type than for the High and Low PLR curve types when more than 10 PAR gradients were measured (Fig. 4). This finding suggests that different designs for PLR curve measurement will have a weak influence on the photosynthetic indicators for the Middle PLR curve type, which indicates that measuring the Middle PLR curve type is more stable if the aim is to compare the photosynthetic characteristics among different trees.

Three optimal measurement designs were suggested for the three types of PLR curves (Table 3) because these designs performed better than the other designs^24,28^, especially in terms of the accuracy of the estimated photosynthetic indicators (AQY, *R*_d_ and *P*_max_, Fig. 5). In addition, we found that the optimal design for the High PLR curve type shifted more towards the high-intensity PAR, which was in contrast to the optimal design for the Low PLR curve type, which shifted more towards the low-intensity PAR.

## Conclusions

PLR curves for a single tree crown generally exhibit obviously vertical variations; thus, the use of a unique measurement design to fit all the PLR curves in a whorl crown does not provide accurate results. The measurement design for the High PLR curve type should shift more towards the high intensity of PAR; however, that for the Low PLR curve type should shift more towards the low intensity of PAR. The accuracies of the estimated AQY, *R*_d_ and *P*_max_ values for the Middle PLR curve type were more stable than those for the High and Low PLR curve types.

## Data Availability

All data generated or analysed during this study can be found at: https://datadryad.org/stash/share/TZhg9SvYVYXDC1nHVNBzT4v5lf57xisydjLZWk1qa-s.
